# Production of a toxic polypeptide as a fusion inside GroEL cavity

**DOI:** 10.1038/s41598-020-78094-8

**Published:** 2020-12-03

**Authors:** Maria S. Yurkova, Elchin G. Sadykhov, Alexey N. Fedorov

**Affiliations:** Bach Institute of Biochemistry, Research Center of Biotechnology of the Russian Academy of Sciences, 33, bld. 2, Leninsky Ave., Moscow, Russia 119071

**Keywords:** Biochemistry, Biotechnology

## Abstract

The system is developed for efficient biosynthetic production of difficult-to-express polypeptides. A target polypeptide is produced fused into *T. thermophilus* GroEL chaperonin polypeptide chain in such a way that it is presented inside the GroEL cavity near the substrate binding surface. Such presentation allows alleviating potential problems of instability, toxicity or hydrophobicity of the fused peptide. Thermostability of thermophilic GroEL can be used for its one-step separation from the host cell proteins by heating. The target polypeptide may be released by any of amino acid-specific chemical treatments. In this study, GroEL was adapted for methionine-specific cleavage with cyanogen bromide by total replacement of methionine residues to facilitate further purification of the target polypeptide. The procedure is simple, robust and easy to scale-up. The capacity of this system to produce difficult-to-express polypeptides is demonstrated by production in bacterial system of one of the most potent antibacterial peptides polyphemusin I.

## Introduction

Peptides are in great demand in different areas, such as research, biopharmaceuticals and industry, and the demand is not completely satisfied by chemical synthesis, which is up to now the main way of producing peptides. Chemical synthesis is a very well developed method, refined to perfection; still, the longer the peptides, the less efficient, more laborious and expensive is their synthesis^1^. For peptides longer than 30 amino acids, the process is sequence dependent and requires individual adjustment^2^, for peptides longer than 35 amino acids, large scale production becomes economically unjustified. Also a problem of growing urgency is that of peptide synthesis producing great amounts of toxic waste^3^. Biosynthetic production looks like an attractive alternative in many ways^4^, but there are some major obstacles in the way of efficiently producing peptides and short proteins in cells. First, there are low expression yields when producing peptides by themselves. To solve this issue, either multiple peptide repeats can be made within a single polypeptide chain, or the peptide sequence can be genetically fused to a soluble carrier protein with a high expression profile^5–10^. A strategy of biosynthetic production of peptides fused to different carrier proteins in bacterial cells has been successfully demonstrated^6,7,11–13^. An alternative possibility is to use a carrier protein designed to direct the fusion to inclusion bodies. This method can be applied to short peptides, intrinsically disordered proteins, and proteins that can be later efficiently refolded^14^. Second, short polypeptides very often don’t have rigid tertiary structure and are subject to intracellular proteolysis. Cell strains deficient in certain proteases may allow alleviation of some of these problems, but, generally speaking, it is difficult to inactivate all proteases as some of them are essential for cell viability and, consequently, the instability of a target peptide in cells may persist. Third, some recombinant peptides and proteins may be toxic for a host cell thus precluding their efficient production. Still, some toxic peptides can be produced in heterologous expression systems^15^, others are biosynthetically produced in microbial cells^4,16,17^, but scale-up in the case of toxic peptides is rarely cost-efficient^17^.

The main purpose of our work was to develop a widely applicable fusion protein system that would be specifically suitable for the production of different peptides which are difficult to express in bacteria for the reasons described above, and that would also allow simple, robust and scalable purification procedures of target polypeptides. The chaperone GroEL is a fitting candidate to be used as a carrier in such a fusion system^18^. It comprises a large particle consisting of two heptamers with a large cavity inside, which accommodates the protein substrate. GroEL is a thoroughly studied chaperone, its structure and functioning have been described in detail^19,20^. Usually one molecule of a protein substrate is presented inside a GroEL ring; as far as the entire particle is concerned, usually negative cooperativity between the two rings is observed in substrate protein binding. The common cavity formed inside the heptamer is sufficient to accommodate a large protein, one of the reports shows binding of 116 kDa beta galactosidase with *E. coli* GroEL^21^.

GroEL is not an easy target to accommodate additional amino acid sequences. Examination of GroEL’s 3D structure suggests that polypeptide extensions at its ends should destabilize the quaternary structure. For this study, the essential issue was to achieve the ability of GroEL to accommodate fused polypeptide inserts within its polypeptide chain without hampering its oligomer structure. The only study that had previously addressed this question was based on the analysis of random transposon inserts in the *groel* gene. It was found that transposon inserts were badly tolerated by the GroEL particle, all of them led to destabilization of the heptamer rings and many mutants were insoluble^22^. Natural limitation of this approach and, consequently, limitation of its conclusions, results from the analysis of a limited number of transposon variants not pre-selected for structure-driven optimal placing of an insert. It is known that random transposon inserts usually inactivate proteins and influence their native structure.

This study describes the development of a GroEL-based fusion carrier, where a target polypeptide is inserted into the chaperonin polypeptide chain so as to be presented inside the cavity near its substrate-binding surface, while the oligomer structure of the particle is not hampered. In this case, the carrier is able to shield the target polypeptide and the cell surroundings from each other. For demonstration of the fact, an extremely toxic antimicrobial polypeptide polyphemusin I was produced in a bacterial cell setting. Modifications introduced in GroEL itself allow streamlining further purification procedures.

## Results

### Development and properties of modified GroEL

Prior to using GroEL as a carrier in a fusion system, some modifications were introduced in it. Several chemical treatments are in use to cleave proteins at a particular amino acid residue or their combination. The most commonly used is CNBr treatment specific for methionine residues, and for the following proof-of-principle study we have made preliminary changes in GroEL to conveniently use this technique. As a first step in engineering an initial framework protein all codons encoding methionine were substituted for ones encoding leucine. GroEL from *T. thermophilus* has six methionine residues in its sequence (initiator methionine is cleaved during polypeptide synthesis as it occurs for almost all proteins); the gene construct encoding GroEL with all methionine residues replaced by leucine residues was obtained by PCR with appropriate primers (see “Methods” section).

**Figure 1:**
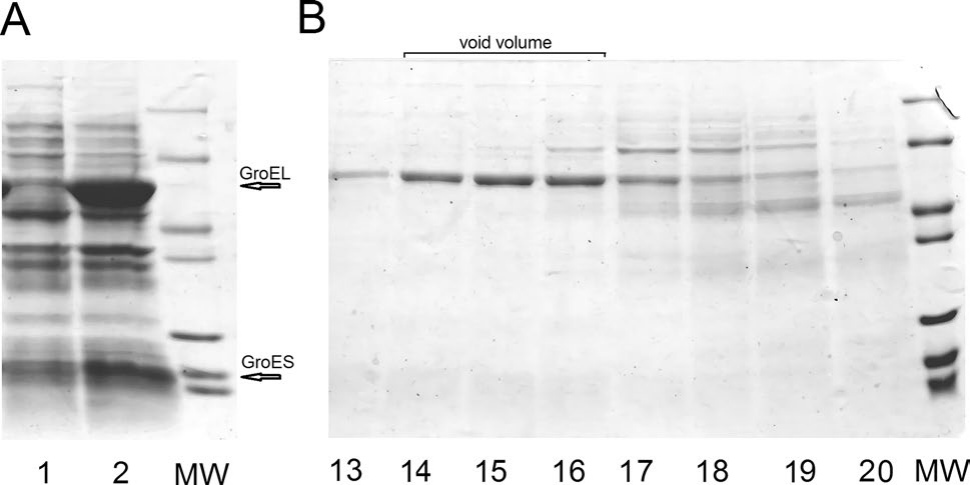
(**A**) Expression of *T. thermophilus* GroES and modified GroEL without methionine residues in the expression bi-cistronic vector pET Duet. Lane 1—before induction, lane 2—after induction. (**B**) Size exclusion chromatography for Met-less GroEL. Lanes 13–20 are numbered according to retention time (min) for corresponding fractions. MW – protein molecular weight markers, 14, 18, 25, 35, 45, 67 and 116 kDa.

The modified gene was cloned into the expression bi-cistronic vector pET Duet together with the gene encoding *T. thermophilus* GroES. It is known from *E. coli* GroEL/ES system that formation of GroEL particles occurs more efficiently when GroEL is co-expressed with GroES. Consequently, the idea was to take this advantage for *T. thermophilus* GroEL fusion system. Both proteins were efficiently synthesized upon expression of this bi-cistronic plasmid (Fig. 1A) and were expressed in the soluble fraction. Though *T. thermophilus* GroEL and GroES (indicated by arrows) can’t be distinguished from their *E. coli* counterparts by size, their production can be assessed as overexpression. Endogenous GroEL and GroES can be seen in the lane 1 on Fig. 1A among other cell proteins as not major bands.

The corresponding GroEL protein was analyzed by size-exclusion chromatography for its oligomer structure on a 300-mm column packed with Sephacryl S-400. In these conditions, predominant part of GroEL was found to have retention time from 13 to 16 min (Fig. 1B). The position of the major GroEL peak corresponds to oligomeric particles as judged against a set of reference proteins (the calibration for the size-exclusion column is represented in Supplementary material, Suppl. Figure 1). The distribution of Met-less GroEL was similar to that of the wild type *T. thermophilus* GroEL expressed in *E. coli* cells, as well as to that of *E. coli* endogenous GroEL/ES complex (data not shown). Thus, the replacement of methionine residues didn’t influence the GroEL quaternary structure.

In the following modifications of the *groel* gene the loop from Ser 199 to Tyr 201 was used to introduce a target polypeptide insert. Short polylinker containing BamHI, HindIII and EcoRI restriction sites was introduced for cloning purposes in the gene between triplets encoding amino acids Ser 199 and Tyr 201. Overall, nucleotides encoding amino acid sequence Gly-Ser-Lys-Leu-Glu-Phe were introduced. This GroEL construct was also cloned into the expression bi-cistronic vector pET Duet together with the gene encoding *T. thermophilus* GroES and, upon expression, its quaternary structure was analyzed by size-exclusion chromatography on a 300-mm column packed with Sephacryl S-400. In this case, too, the complex retained its oligomeric structure intact (data not shown). Both GroEL variants, Met-less and Met-less with introduced polylinker, were tested for thermostability as compared with the wild type *T. thermophilus* GroEL expressed in *E. coli* cells. The test consisted in heating soluble cell proteins in bulk at 65 °C for 5 min and then separating aggregated ones by centrifugation. The result was assessed by electrophoresis of normalized samples and showed that all three GroEL variants remained soluble after the treatment and thus could be easily separated from mesophilic host proteins.

### Expression and properties of GroEL/polyphemusin I fusion

**Figure 2:**
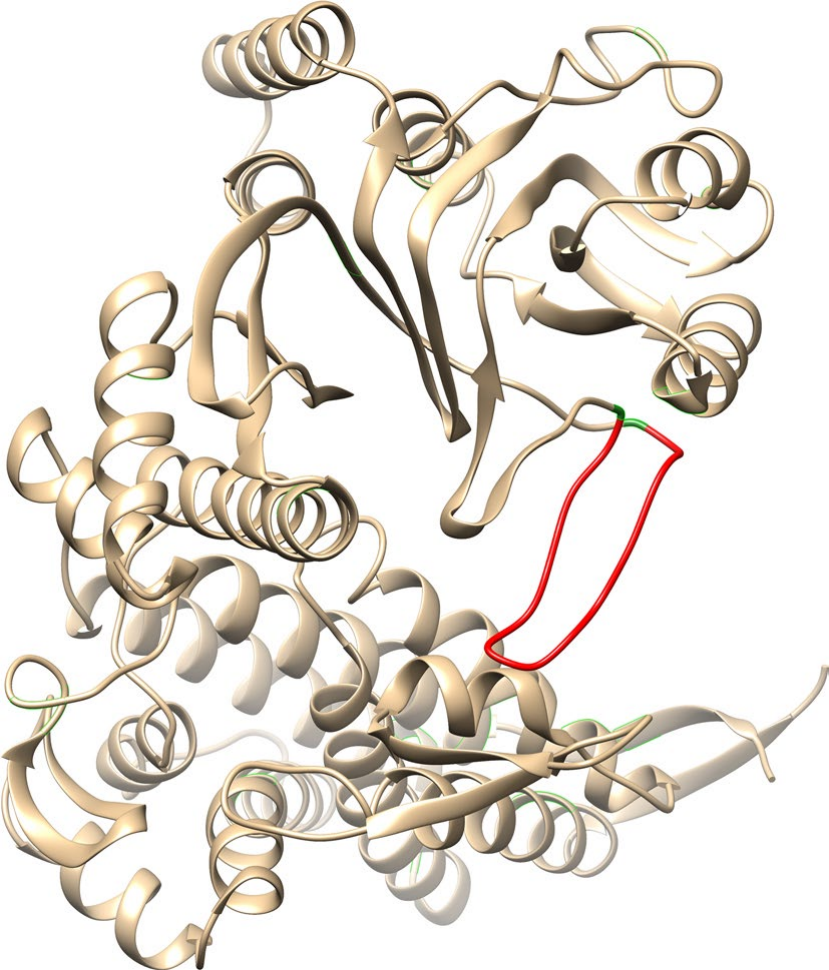
The place of fused polyphemusin I (shown in red) in GroEL polypeptide chain. GroEL is shown as a monomer. To illustrate the position of inserted polyphemusin I, the published structure of *T. thermophilus* GroEL available in pdb-bank at http://www.rcsb.org/structure/4V4O was used. For manipulations with the structure the program Chimera 1.13.1 was used.

The gene encoding polyphemusin I, with added codons for two Met residues immediately flanking the insert to allow further chemical cleavage of the target peptide, and restriction sites BamHI/EcoRI was chemically synthesized. Codon usage of the insert was adjusted for codon usage in *E.coli* (for details, see “Methods” section). The insert was cloned into the modified GroEL gene (Met-less with polylinker) using restriction sites BamHI/EcoRI. Figure 2 illustrates the suggested position of the insert.

**Figure 3:**
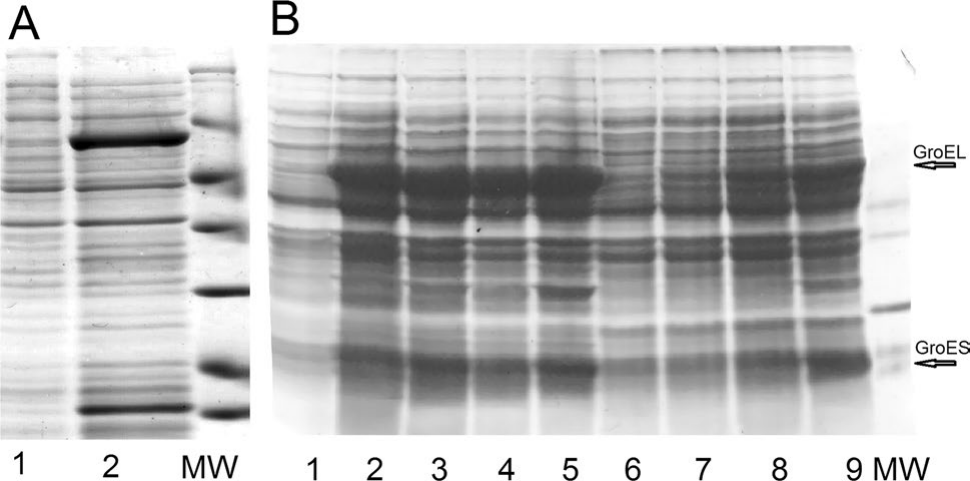
(**A**) Expression of GroES and GroEL fusion with polyphemusin I in pLysE cells. Lane 1—before induction, lane 2—after induction. (**B**) Expression of GroES and GroEL fusion with polyphemusin I in two different ways, IPTG induction vs autoinduction. For IPTG induction: lane 1—before induction, lane 2—1 h after induction, lane 3—2 h after induction, lane 4—3 h after induction, lane 5—4 h after induction; for autoinduction: lane 6—4 h incubation, lane 7—5 h incubation, lane 8—6 h incubation, and lane 9—overnight incubation.

The resulting construct was expressed along with GroES in BL21(DE3) pLysE cells, which are specifically suited for the expression of toxic recombinant proteins, and demonstrated high expression levels (Fig. 3A).

**Figure 4:**
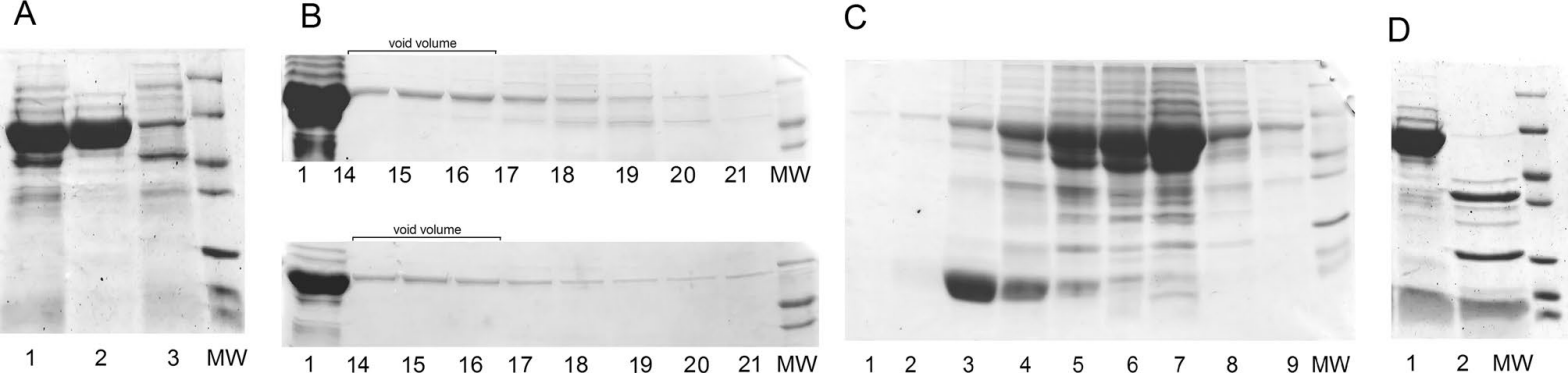
(**A**) Separation of GroEL fusion by heating. Lane 1—cell lysate before heating, lane 2—supernatant after heating, lane 3—pellet after heating. (**B**) Size exclusion chromatography for GroEL-polyphemusin I fusion, upper panel—before heating, lower panel—after heating. For both panels: lane 1—initial material loaded onto the column; 14–21—retention time (in minutes) for corresponding fractions. (**C**) Separation of GroEL-polyphemusin I fusion on C4 column. Lanes 1—9—fractions from C4 column. (**D**) CNBr treatment of GroEL-polyphemusin I fusion, lane 1—before, lane 2—after treatment.

Different ways of expression, autoinduction versus IPTG induction, were tested, and IPTG induction proved to give best results (Fig. 3B); for details, see “Methods” section. Identity of the produced target as intact polyphemusin I without any amino acid substitutions was confirmed (see “Confirmation of polyphemusin I identity” section). With IPTG induction, the average production of modified GroEL was 200 mg from 1 L of culture, and as polyphemusin I constitutes 3.8% of the whole construct, that gives average theoretical production of polyphemusin I 7.6 mg per liter of culture. In practice, we were able to quantify the yield of polyphemusin I after completing the purification procedure, and it ranged from 4.0 to 4.8 mg per liter of culture. In general, the yield of biosynthetic production of toxic peptide depends on the nature of the peptide and is reported to range between 0.8 mg/ml for Hepcidins and 35.8 mg/ml for Plecstasin^17^.To assess the thermostability of obtained fusion, the cell lysate was subjected to heating at 65 °C for 5 min. GroEL fusion entirely remained in solution after centrifugation of aggregated cell proteins (see Fig. 4A).

The oligomer structure of GroEL-polyphemusin I fusion was analyzed by SEC on a 300-mm column packed with Sephacryl S-400 both before and after the heating procedure. The modified GroEL containing fused polyphemusin I was eluted as oligomeric particles, and the elution profile remained unchanged even after heating at 65 °C for 5 min (compare Fig. 4B upper and lower panels). In both cases, the particle was eluted in exclusion volume, showing the same retention time as the Met-less GroEL (Fig. 1B).

### Purification of polyphemusin I

Heating at 65 °C for 10 min with following separation of aggregated host proteins by centrifugation was used as a first step of purification. Prior to chemical treatment with CNBr, GroEL-polyphemusin I fusion was subjected to reverse phase chromatography on C4 resin in conditions that led to partial separation of GroES (Fig. 4C). The GroEL-polyphemusin I fusion was eluted from C4 column in a volatile buffer. After that, the fractions of interest were combined, lyophilized and re-dissolved to be subjected to CNBr treatment according to manufacturer’s instructions (see “Methods” section). The reaction was complete and yielded two large GroEL fragments, the remaining GroES and polyphemusin I (Fig. 4D). After CNBr treatment, the reaction was lyophilized, and peptides re-dissolved in water/0.1% TFA/5% acetonitrile for loading onto C18 column. It should be noted that, while peptides readily dissolve in water in acidic conditions, large fragments of GroEL do not. Thus, the reverse phase chromatography on C18 column served for separation of polyphemusin I from other peptides.

### Confirmation of polyphemusin I identity

**Figure 5:**
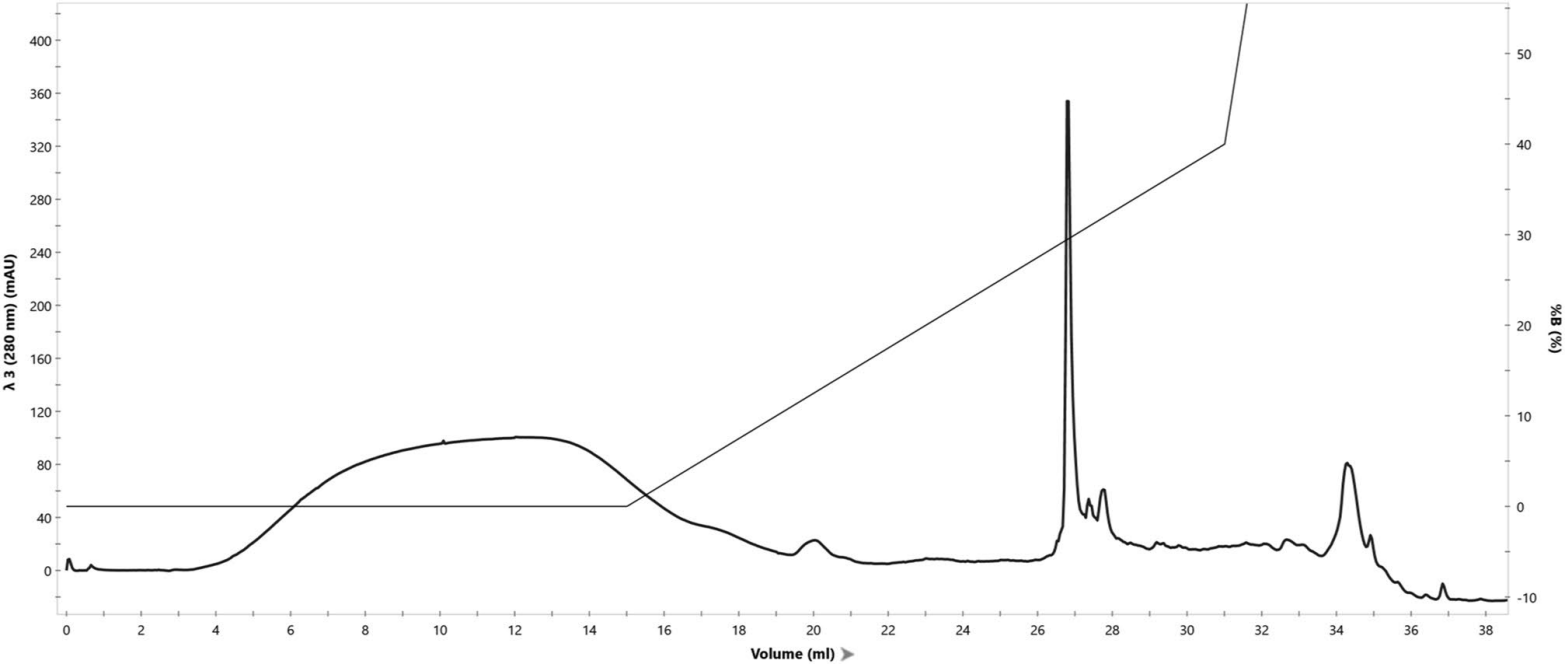
Reverse phase chromatography of obtained polyphemusin I on C18 column. The peptide was eluted in a gradient of acetonitrile in water in the presence of 0.1% TFA between 31 and 32% acetonitrile, which coincides with the retention of chemically synthesized peptide.

The identity of polyphemusin I purified from GroEL fusion was confirmed by its chromatographic properties (Fig. 5), by mass spectrometry and by its bacteriostatic action against wild-type *E. coli* UB1005. The purified polyphemusin I was eluted as a complex peak due to different oxidation status of its four Cys residues which in the native polypeptide form two disulfide bonds. The chromatogram of chemically synthesized polyphemusin I, for comparison, is shown on Suppl. Figure 2.

**Figure 6:**
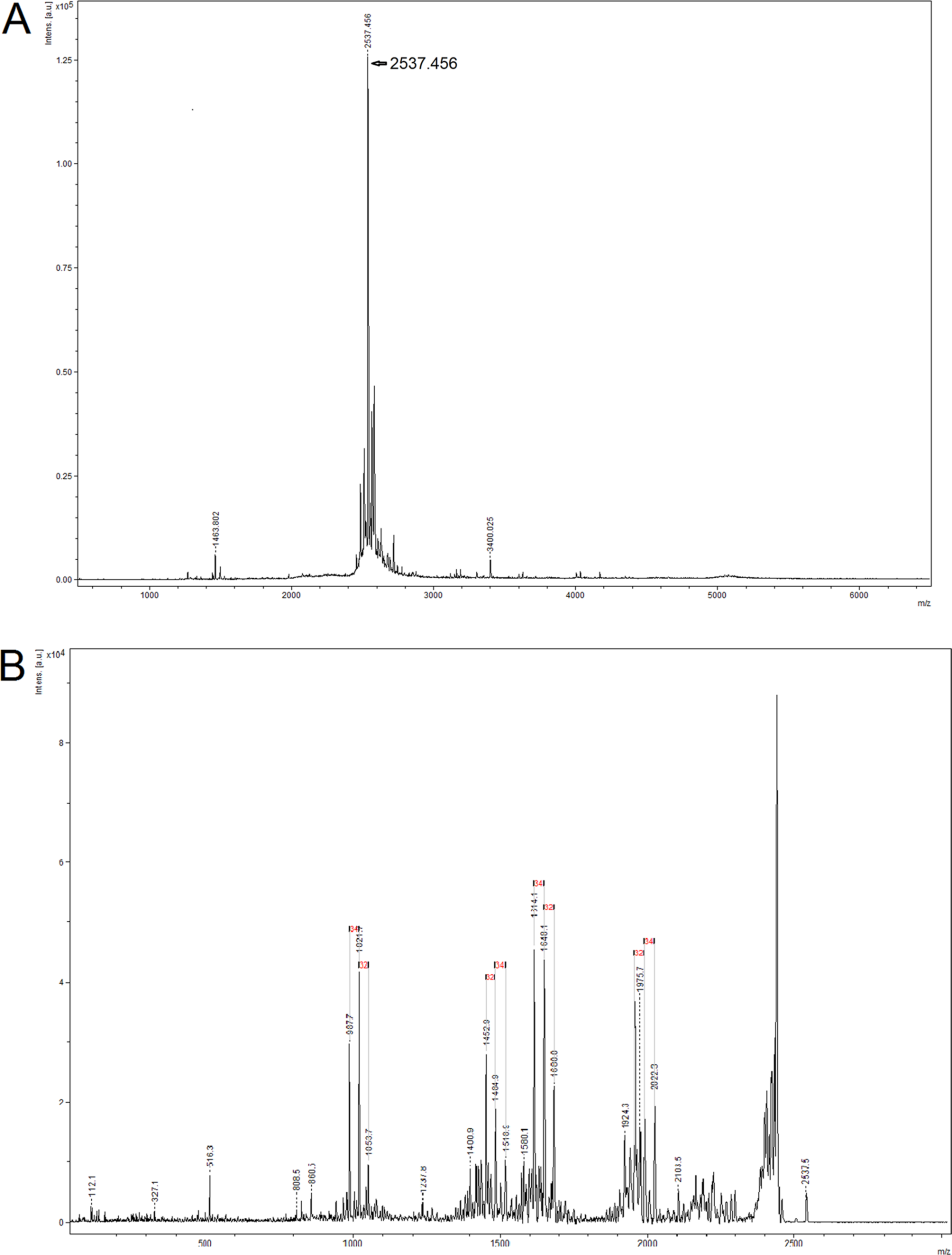
Mass-spec (**A**) and ms–ms (**B**) for polyphemusin I purified from GroEL fusion.

The identity of obtained polyphemusin I was further proved by mass-spectrometry (Fig. 6). The molecular mass of the main peak (Fig. 6A) corresponds to fully oxidized form of polyphemusin I (depending on oxidation state of cysteine residues, the expected molecular mass ranges between 2537 and 2541 Da); ms–ms (Fig. 6B) confirms the presence of four cysteine residues.

Obtained polyphemusin I, being a potential antibacterial agent, was tested for bacteriostatic activity after its cleavage from GroEL fusion and purification. Its minimal inhibitory concentration (MIC) against wild-type *E. coli* UB1005 was determined in three independent experiments in parallel with the chemically synthesized analog (see “Methods” section). MIC values in both cases were shown to be 3 μg/ml (1.25 µM), which is consistent with the data of other authors^23,24^.

## Discussion

The goal of this study has been to make a carrier for a protein fusion system that would simultaneously serve several purposes ultimately directed at a robust and cost-efficient production of diverse peptides including difficult-to-produce peptides, e.g. proteolytically labile and toxic target peptides. The GroEL chaperone has been chosen as a carrier protein to provide several key benefits for the system. A choice of the particular GroEL for making the carrier for fusion system was based on several considerations. GroEL is essential for bacterial cell viability. Using host bacterial GroEL as a basis for fusions would on all counts make it nonfunctional or poorly functional for the folding of host proteins, thus hampering cell viability. To avoid these complications, we have chosen exogenous GroEL for making fusion gene constructs for the expression in *E. coli* cells. A variety of GroEL counterparts could have been used for this matter, particular choice of GroEL from *T. thermophilus* was made because its tertiary structure is known^25^ and because it is thermostable, which can be utilized in the purification process. Structurally, type I GroEL is a double-ring 14-mer particle with a single-ring heptamer forming a common cavity involved in the binding of substrate proteins. The structure is further stabilized by a heptamer ring of co-chaperone GroES. The GroEL monomer consists of three domains. The one is equatorial domain, primarily responsible for inter-subunit contacts in a heptamer and between two heptamer rings of the particle; the intermediate domain also participates in inter-subunit contacts in a heptamer and is largely involved in conformational rearrangements of GroEL particle; the apical domain is a principal site for binding of substrate polypeptides and co-chaperone GroES. The apical domain has predominantly alpha helical secondary structure, its surface facing cavity consists of loosely packed loops, and these loops serve as natural recipients to accommodate target polypeptides. In our structure-driven approach, appropriate structural elements on the internal surface of the GroEL heptamer were chosen in such a way that polypeptide inserts incorporated there would have no impact on GroEL oligomer structure and, additionally, an insert would be allowed to bind to the substrate-binding surface. Such binding may additionally stabilize labile peptides and alleviate their toxic propensities, if present. Based on these considerations, a small loop from Ser 199 to Tyr 201 in the apical domain was used to introduce a target polypeptide insert. To streamline further purification procedures, all six methionine residues in GroEL were substituted for leucine ones. Thus modified GroEL co-expressed with GroES retained the thermostability and quaternary structure of the original particle. This is a valuable feature in a carrier for fusion system, because non-chromatographic methods of purification, if applicable, are preferred at scaling-up as cost-efficient^17^. Another option is to use tangential filtration as the first step of purification to separate the particle from the rest of proteins, taking advantage of its size, which is about 10^6^ Da.

Polyphemusin I, one of the most toxic antimicrobial peptides, has been chosen as a target peptide to demonstrate the capacity of the GroEL-based fusion system in the production of difficult-to-express peptides. Polyphemusin I is an 18 amino acid peptide, represented by an amphipathic beta-hairpin connected by a type IV beta-turn^26^. Its minimal inhibitory concentration for bacteria was reported to be 0.5–25 mcg/ml^23,24,26,27^.

To authors’ knowledge, there is one work about biosynthetic production of polyphemusin I fused with maltose-binding protein as a leader^24^, but in our hands the attempts to produce polyphemusin I fused to thioredoxin, or as GroEL-polyphemusin I fusion without GroES in BL21(DE3) pLysE cells, or as GroEL-polyphemusin I fusion along with GroES in cells BL21(DE3) without tightly regulated promoter were equally unsuccessful and led either to cell death or production of heavily mutated polyphemusin I (the unsuccessful fusion constructs with polyphemusin I are listed in Suppementary material, Suppl. Table 1). This indicates that formation of stable GroEL particles achieved in the presence of GroES is required to efficiently shield the toxic target from its action inside the bacterial cell. On the other hand, the successful expression of GroEL-polyphemusin I fusion indirectly indicates the fact of formation and stability of the correct GroEL/ES quaternary structure, which was further confirmed by size-exclusion chromatography.

Thus, as a proof of principle the described system allowed obtaining a highly toxic peptide in bacteria. In this study, GroEL from *T. thermophilus* was reengineered by substituting all Met residues for the use of CNBr cleavage to simplify purification of a target peptide. Similar optimization of GroEL can be done for the use of other chemical treatments: formic acid is specific for X/Y sequence; only one such amino acid sequence is present in *T. thermophilus* GroEL and can readily be changed.

Summing up, it can be concluded that the two-level protection which GroEL-based fusion may provide for a target peptide allows biosynthetic production of most difficult-to-express peptides. Purification procedures which may be applied to obtain a target peptide are very simple, robust and easily scalable.

## Methods

### Gene and plasmid construction

#### Construction of Met-less T. thermophilus GroEL

The sequence of *T. thermophilus* GroEL contained five methionine residues, which were replaced by PCR using following pairs of primers:

- TT-1 lo: 5′CCACCTTCTCCAGGGCGTCGGCAATCAGCTTG3′,
- TT-1 up: 5′GATTGCCGACGCCCTGGAGAAGGTGGGGAAGG3′;
- TT-2 lo: 5′AGGACCGCTTCCAGCGTCTCGGGGTTGGTGAC3′,
- TT-2 up: 5′CAACCCCGAGACGCTGGAAGCGGTCCTCGAGG3′;
- TT-3 lo: 5′TGTCCTTGAGCAGCTCCTTCCTGCGGTCACCGAAG3′,
- TT-3 up: 5′ACCGCAGGAAGGAGCTGCTCAAGGACATCGCGG3′;
- TT-4 lo: 5′CCGGCCCAGCAGGGAGAGGGTGGCGTTCTC3′,
- TT-4 up: 5′GCCACCCTCTCCCTGCTGGGCCGGGCCGAG3′;
- TT-5 lo: 5′CGCCTCCACCAGGTCCACGAACTCCCCGGTG3′,
- TT-5 up: 5′GGAGTTCGTGGACCTGGTGGAGGCGGGCATCG3'.

The sixth methionine residue was replaced in the PCR with the following primers, forward primer TT-f-NdeI: 5′GGGAATTCCATATGGCGAAGATCCTGGTGTTTGACG3′ and reverse primer TT-r-BglII no Met: 5′GGAAGATCTTTAGAAATCCAGGTCCCCGGCGC3′, which produced methionine-less GroEL ending with *TAA* stop codon and flanked by recognition sites for NdeI and BglII (underlined). This fragment was digested with NdeI and BglII and cloned into the second polylinker of expression bi-cistronic vector pET Duet together with the gene encoding *T.thermophilus* GroES yielding expression vector pGroEL/ES.

#### Cloning of GroES

*T. thermophilus* GroES was amplified using forward primer f-NcoI CATGCCATGGCCGCGGAGGTGAAGACG and reverse primer r-NotI TTTTCCTTTTGCGGCCGCTTACTGCAGGACCGCAAGCAGGTCG, containing restriction sites for NcoI and NotI (underlined), respectively, and *T. thermophilus* genome as a template. Obtained PCR fragment was digested with NcoI and NotI restricition enzymes and cloned into the first polylinker of expression bi-cistronic vector pET Duet together with the gene encoding *T.thermophilus* methionine-less GroEL yielding expression vector pGroEL/ES.

#### Introducing the polylinker into GroEL gene construct

Short polylinker containing BamHI, HindIII and EcoRI was introduced for cloning purposes in the gene between triplets encoding amino acids Ser 199 and Tyr 201. At the first step, the two segments of the desired gene construct were separately amplified using methionine-less GroEL sequence as a template. One segment was amplified using forward primer TT-f-NdeI: 5′GGGAATTCCATATGGCGAAGATCCTGGTGTTTGACG3′ and reverse primer TT-r-201: 5′GGTGACGAATTCAAGCTTGGATCCGATGTACCCCTTGTCAAACTGGTA3′; the other one using forward primer TT-f-201: 5′GGATCCAAGCTTGAATTCGTCACCAACCCCGAGAC3′ and reverse primer TT-r-BglII no Met 5′GGAAGATCTTTAGAAATCCAGGTCCCCGGCGC3’.

Combined amplification using purified fragments obtained at the previous step as templates and two primers, TT-f-NdeI and TT-r-BglII no Met, produced nucleotide sequence of methionine-less GroEL with polylinker. This fragment was digested with NdeI and BglII restriction enzymes and cloned into the second polylinker of the expression bi-cistronic vector pET Duet together with the gene encoding *T.thermophilus* GroES yielding expression vector ploop/ES.

#### Cloning of polyphemusin I

Polyphemusin I (http://www.ncbi.nlm.nih.gov/protein/P14215) sequence CGTCGTTGGTGCTTTCGTGTGTGCTATCGTGGCTTTTGCTATCGTAAATGCCGT was adjusted for codon usage in *E.coli* (changes shown in bold letters below), and codons for two Met residues immediately flanking the insert to allow further chemical cleavage of the target peptide were added: *ATG*CGTCG**C**TGGTGCTTTCGTGTGTGCTATCG**C**GGCTTTTGCTATCGTAAATGCCG**CATG**. This variant of polyphemusin I was fused from two primers, Phemu-for: 5′**GATCC**ATGCGTCGCTGGTGCTTTCGTGTGTGCTATCGCGGCTTTTGCTATCGTAAATGCCGCATG**G**3′ and Phemu-rev: 5′**AATTC**CATGCGGCATTTACGATAGCAAAAGCCGCGATAGCACACACGAAAGCACCAGCGACGCAT**G**3′. The primers were designed in such a way that the resulting polyphemusin I had ready sticky ends (shown in bold letters) for cloning into BamHI/EcoRI sites of the polylinker introduced into GroEL gene. The bi-cictronic vector ploop/ES was digestetd with BamHI and EcoRI restriction enzymes, and polyphemusin I was cloned into it.

All the results of cloning procedures were confirmed by sequencing.

### Expression of the construct and purification of target polypeptide

#### Expression in the presence of IPTG or by autoinduction

The construct was expressed in BL21(DE3) pLysE cells grown in LB medium at 37 °C, at OD_600_ 0.4 IPTG was added to the final concentration of 0.4 mM, and after 3 h the cells were collected by centrifugation. Alternatively, autoinduction was conducted at 37 °C overnight in TB (12 g tryptone, 24 g yeast extract per 1 L) containing P × 20 (1 M Na_2_HPO_4_, 1 M KH_2_PO_4_, 0.5 M (NH_4_)_2_SO_4_); P × 50 (25% glycerol, 2.5% glucose, 10% alpha-lactose monohydrate) and trace elements, final concentrations: 50 μM FeCl_3_, 10 μM MnCl_2_, 2 μM CoCl_2_, 2 μM NiCl_2_, 2 μM Na_2_SeO_3_, 20 μM CaCl_2_, 10 μM ZnSO_4_, 2 μM CuCl_2_, 2 μM Na_2_MoO_4_, 2 μM H_3_BO_3_; 2 mM MgSO_4_; ampicillin 200 μg/ml. After overnight autoinduction, the cells were collected by centrifugation. In all cases, collected cells were lysed by sonication on ice in the presence of 4-(2-aminoethyl)benzenesulfonyl fluoride hydrochloride, a protease inhibitor, and soluble proteins separated from cell debris by centrifugation. GroEL/ES complex was expressed in a soluble state.

#### Purification by heating from host proteins

Heating of the cell lysate at 65 °C for 5 min was performed in water bath and followed by centrifugation at 13,000*g* for 15 min.

*Separation of GroEL on C4 column* was conducted on Symmetry300 C4 (3.9 × 150 mm, 5 μm, Waters) column in water/acetonitrile system in the presence of 0.1% TFA. Proteins were eluted in the gradient of acetonitrile. Fractions containing GroEL fusion with polyphemusin I were lyophilized prior to CNBr treatment.

#### CNBr treatment

The reaction was conducted overnight at room temperature in 70% formic acid in water, CNBr concentration was 50 mg/ml. The reaction was quenched by freezing at − 70 °C; the samples were lyophilized and re-dissolved in appropriate solutions for reverse phase chromatography on C18 column or for electrophoresis.

*Purification of polyphemusin I on C18* was conducted on Symmetry300 C18 (4.6 × 150 mm, 5 μm, Waters) column. Samples, lyophilized after CNBr treatment, were re-dissolved in 5% acetonitlile/0.1% TFA in water. In these conditions, large fragments of GroEL do not dissolve, while peptides readily do. Peptides were separated from each other in isocratic conditions at 20% acetonitrile/0.1% TFA.

### Characterization of expressed protein and target polypeptide

*Size exclusion chromatography* of the GroEL/ES complex was conducted on Tricorn 10/300 column packed with Sephacryl S400, in PBS, flow rate 1 ml/min. The column was calibrated with Gel Filtration Calibration Kit HMW (GE Healthcare). Blue dextran (about 2000 kDa, exclusion volume), which corresponds in our experiments to 14-mer GroEL/ES, was eluted at 16 min, BSA (MW 67 kDa, corresponds to the GroEL monomer) at 19.5 min, and salts after 22 min.

*Reverse phase chromatography of polyphemusin I on C18* was conducted on Symmetry300 C18 (4.6 × 150 mm, 5 μm, Waters) column, program 0–40% acetonitrile (in the presence of 0.1% TFA) in 15 ml, flow rate 0.4 ml/min. Polyphemusin I is eluted between 31 and 32% acetonitrile.

*MALDI-TOF/TOF mass-spectrometry* Spectra of the samples were obtained at MALDI-TOF/TOF mass spectrometer with laser desorption/ionization UltrafleXtreme (Bruker, Germany), equipped with UV laser in the mode of positive ions with the use of reflectron. Spectra were obtained in the mass range 500–6500 m/z, by choosing the optimal laser power to achieve the best resolution.

#### MIC determination

Minimal inhibitory concentration (MIC) was defined as the lowest peptide concentration at which the growth of wild-type *E. coli* strain UB1005 was completely quenched as observed after an overnight incubation at 37°C^28,29^. MIC values were determined for polyphemusin I purified from GroEL fusion and for chemically synthesized polyphemusin I (rrwcfrvcyrgfcyrkcr) as a control. Both samples were dissolved in phosphate buffered saline (10 mM KH_2_PO_4_ pH 7.4, 140 mM NaCl), their initial concentration was measured at OD_280_. MIC values were determined using the broth macro-dilution method in Mueller–Hinton medium, with initial cell concentration 5 × 10^5^ cells/ml, to which both samples of polyphemusin I in two-fold dilutions were added. Each concentration point was taken in triplicate; results represent the mean value of three independent experiments.

## Supplementary information

Supplementary Legends.

Supplementary Figure S1.

Supplementary Figure S2.

Supplementary Table S1.
