# Supplementary material for: Production of a toxic polypeptide as a fusion inside GroEL cavity
Source: Sci Rep. 2020 Dec 3;10:21024. doi: 10.1038/s41598-020-78094-8 (PMC7713045; doi:10.1038/s41598-020-78094-8)
Supplement: Supplementary file 1 — Supplementary Legends. [file 41598_2020_78094_MOESM1_ESM.docx]

Suppl. Fig. 1. Calibration curve for size exclusion 300 mm column packed with Sephacril S-400. Retention time (in minutes) for reference proteins, kDa: blue dextran 1000, ferritin 400, bovine serum albumin 67, lysozyme 14.

Suppl. Fig. 2. Reverse phase chromatography of chemically synthesized polyphemusin I on C18 column. The peptide was eluted in a gradient of acetonitrile in water in the presence of 0.1% TFA between 31% and 32% acetonitrile.
