# Supplementary figures and images for: Production of a toxic polypeptide as a fusion inside GroEL cavity
Source: Sci Rep. 2020 Dec 3;10:21024. doi: 10.1038/s41598-020-78094-8 (PMC7713045; doi:10.1038/s41598-020-78094-8)

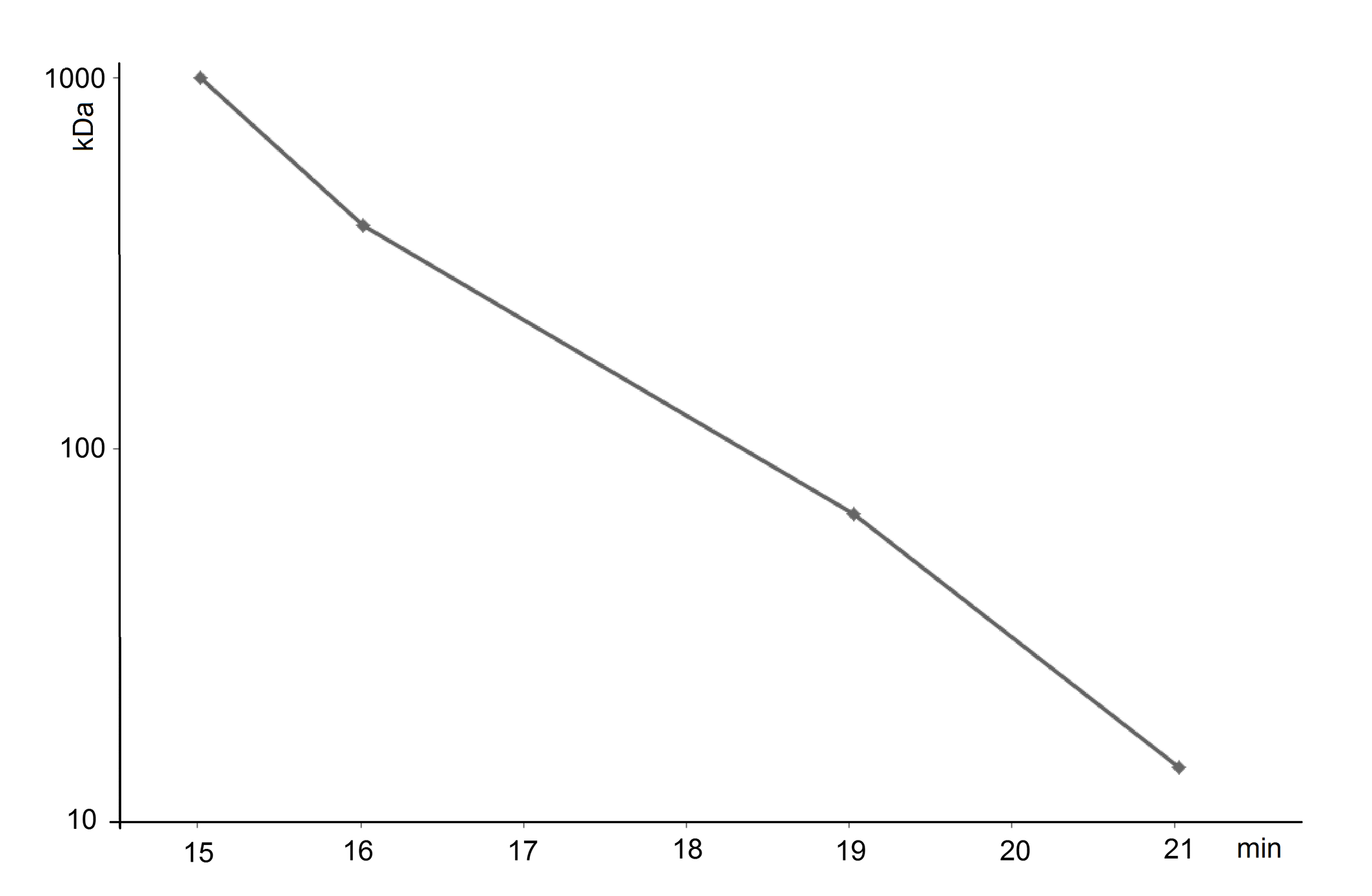

Supplement: Supplementary file 2 — Supplementary Figure S1. [file 41598_2020_78094_MOESM2_ESM.tif]

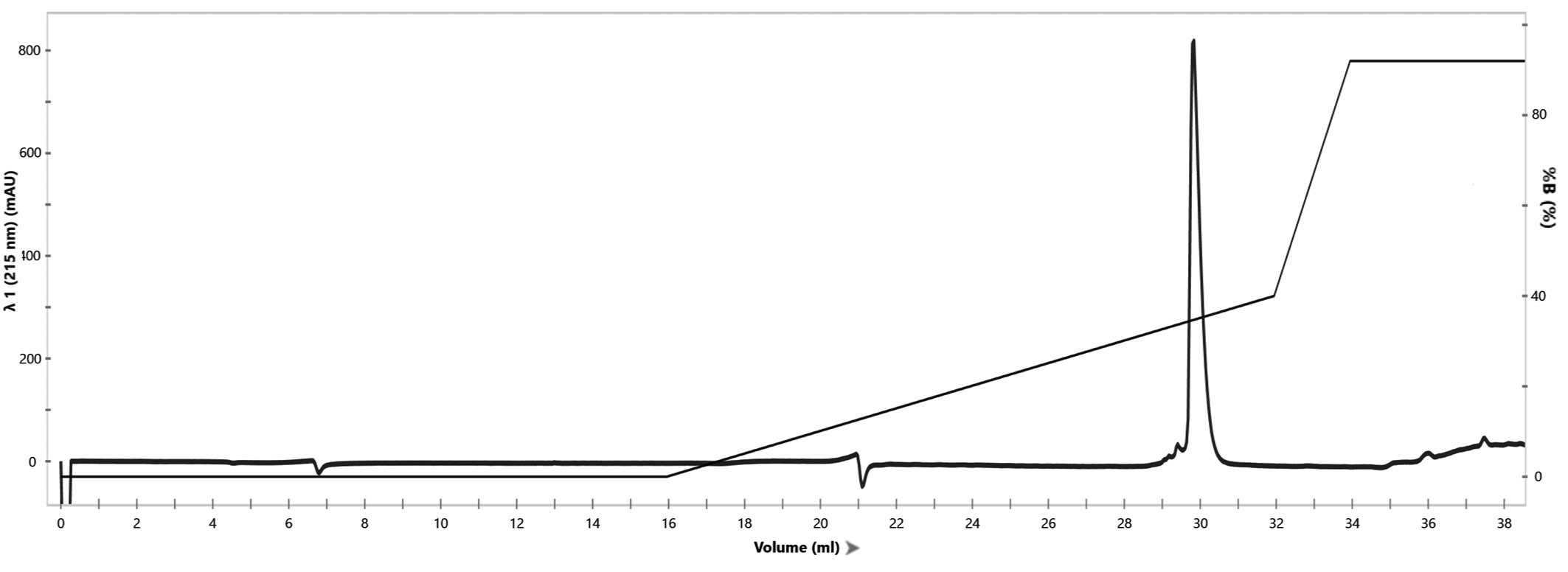

Supplement: Supplementary file 3 — Supplementary Figure S2. [file 41598_2020_78094_MOESM3_ESM.tif]
