# Supplementary material for: Production of a toxic polypeptide as a fusion inside GroEL cavity
Source: Sci Rep. 2020 Dec 3;10:21024. doi: 10.1038/s41598-020-78094-8 (PMC7713045; doi:10.1038/s41598-020-78094-8)
Supplement: Supplementary file 4 — Supplementary Table S1. [file 41598_2020_78094_MOESM4_ESM.docx]

Table I. Biosynthetic production of polyphemusin I in different constructs.

| Construct | Plasmid | Cell line | Cell growth | Expression |
| --- | --- | --- | --- | --- |
| Thioredoxin – polyphemusin I* | pET23a | BL21(DE3) | - | - |
| GroEL (loop) – polyphemusin I** | pET23a | BL21(DE3) | - | - |
| GroEL (loop) – polyphemusin I | pET23a | BL21(DE3) pLysE | - | - |
| GroEL (loop) – polyphemusin I / GroES | pET-Duet | BL21(DE3) | ± | ± |
| GroEL (loop) – polyphemusin I / GroES | pET-Duet | BL21(DE3) pLysE | + | + |

*amino acid sequence of the construct Thioredoxin – polyphemusin I, the linker is shown in italics, polyphemusin I in bold MSDKIIHLTDDSFDTDVLKADGAILVDFWAEWCGPCKMIAPILDEIADEYQGKLTVAKLNIDQNPGTAPKYGIRGIPTLLLFKNGEVAATKVGALSKGQLKEFLDANLA*SGSGAGDDDDK***RRWCFRVCYRGFCYRKCR**

**amino acid sequence of the construct GroEL (loop) – polyphemusin I, polyphemusin I is shown in bold, flanking methionines are shown in italics, amino acid residues corresponding to BamHI and EcoRI restriction sites are shown in bold italics AKILVFDEAARRALERGVNAVANAVKVTLGPRGRNVVLEKKFGSPTITKDGVTVAKEVELEDHLENIGAQLLKEVASKTNDVAGDGTTTATVLAQAIVREGLKNVAAGANPLALKRGIEKAVEAAVEKIKALAIPVEDRKAIEEVATISANDPEVGKLIADAMEKVGKEGIITVEESKSLETELKFVEGYQFDKGYIS***GS****M***RRWCFRVCYRGFCYRKCR***M****EF***YFVTNPETMEAVLEDAFILIVEKKVSNVRELLPILEQVAQTGKPLLIIAEDVEGEALATLVVNKLRGTLSVAAVKAPGFGDRRKEMLKDIAAVTGGTVISEELGFKLENATLSMLGRAERVRITKDETTIVGGKGKKEDIEARINGIKKELETTDSEYAREKLQERLAKLAGGVAVIRVGAATETELKEKKHRFEDALNATRAAVEEGIVPGGGVTLLRAISAVEELIKKLEGDEATGAKIVRRALEEPARQIAENAGYEGSVIVQQILAETKNPRYGFNAATGEFVDMVEAGIVDPAKVTRSALQNAASIGALILTTEAVVAEKPEKKESTPASAGAGDMDF
